# A ruptured aneurysm in the vasa corona at the craniocervical junction with dysgenesis of the posterior inferior cerebellar artery

**DOI:** 10.1259/bjrcr.20160004

**Published:** 2016-05-08

**Authors:** Katsuhiro Mizutani, Takenori Akiyama, Dai Kamamoto, Hideaki Nagashima, Kazunari Yoshida

**Affiliations:** Department of Neurosurgery, Keio University School of Medicine, Tokyo, Japan

## Abstract

This paper reports the case of a ruptured aneurysm in the vasa corona at the craniocervical junction with dysgenesis of the posterior inferior cerebellar artery (PICA). Dysgenesis of the proximal PICA in the present case caused the development of a pial anastomosis in the vasa corona, resulting in the formation of an aneurysm because of increased haemodynamic stress at the vasa corona. The aneurysm was successfully treated with transarterial coil embolization. The clinical entity in the present case is extremely rare and it is important to consider an aberrant vascular anomaly as the cause of an isolated spinal aneurysm.

## Summary

This paper reports the case of a ruptured aneurysm in the vasa corona at the craniocervical junction with dysgenesis of the posterior inferior cerebellar artery (PICA). Dysgenesis of the proximal PICA in the present case caused the development of a pial anastomosis in the vasa corona, resulting in the formation of an aneurysm because of increased haemodynamic stress at the vasa corona. The aneurysm was successfully treated with transarterial coil embolization. The clinical entity in the present case is extremely rare and it is important to consider an aberrant vascular anomaly as the cause of an isolated spinal aneurysm.

## Clinical presentation

A 42-year-old male experienced sudden posterior cervical pain and visited a nearby hospital. He had no history of trauma or systemic inflammatory diseases. His neurological examination indicated only diplopia in all directions of gaze in addition to the cervical pain.

## Imaging findings

**Figure 1.**
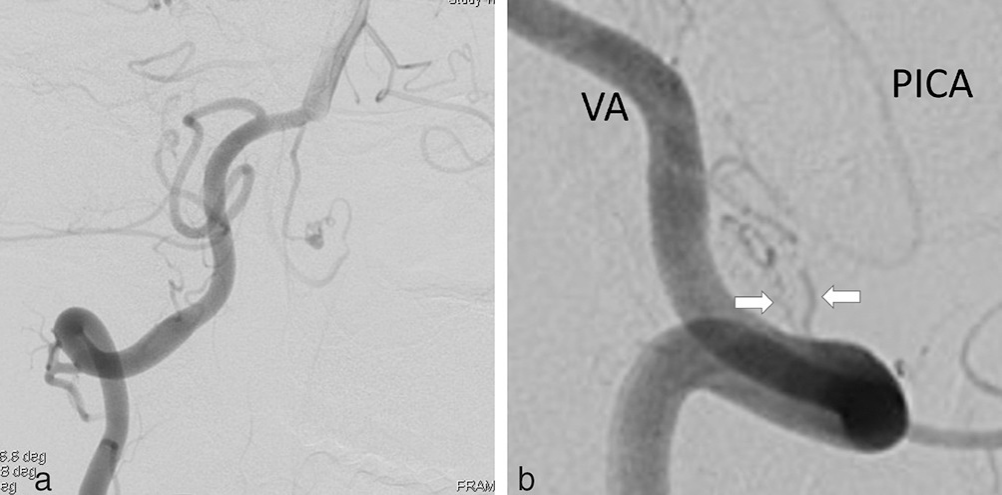
Angiography of the right VA (a) (right anterior oblique 26 and cranial 10 view) and the left VA (b) (lateral view). There are two anterior spinal arteries originating from the right VA immediately proximal to the union. The dilated vasa corona diverged from the left anterior spinal artery, along the left anterior surface of the medulla. The distal end of the vasa corona is dorsally located and is continuous with the left PICA. Left vertebral angiography demonstrates the left PICA but there is no robust connection between the left PICA and the left VA. They are connected together by small tortuous arteries (white arrows). PICA, posterior inferior cerebellar artery; VA, vertebral artery.

**Figure 2.**
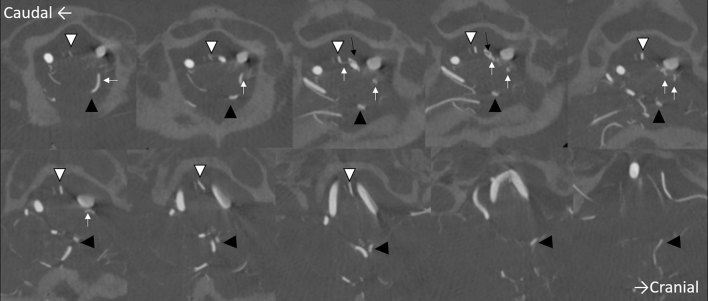
Axial three-dimensional rotational angiography of the left vertebral artery. The aneurysm (small black arrows) is located in front of the medulla at the midline. The dilated vasa corona (small white arrows) distal to the aneurysm is tortuous and continuous with the left posterior inferior cerebellar artery (black arrowheads). The anterior spinal artery is indicated by the white arrowheads.

CT scan demonstrated diffuse subarachnoid haemorrhage (SAH) from the craniocervical junction to the suprasellar cistern. Digital subtraction angiography findings did not reveal intracranial aneurysms in the major arteries, but angiography of the right vertebral artery revealed a small saccular aneurysm on the vasa corona at the craniocervical junction. The patient had two anterior spinal arteries originating from the right vertebral artery just proximal to the vertebral union (Figure 1a). Both anterior spinal arteries ran caudally along the anterior side of the medulla. The dilated vasa corona, where the aneurysm was located, diverged from the left anterior spinal artery and ran along the left anterior and lateral surfaces of the medulla and was continuous with the left PICA on the posterolateral surface of the medulla. The left PICA had a weak, and not a normal robust connection with the left vertebral artery *via* two tiny tortuous arteries (Figure 1b). Three-dimensional rotational angiography revealed in detail the location of the aneurysm and the connections between the anterior spinal artery, the vasa corona, the aneurysm and the left PICA (Figure 2). There were no arteriovenous shunts, such as arteriovenous malformation or dural arteriovenous fistula, which could be a secondary cause of the aneurysms.

## Treatment

**Figure 3.**
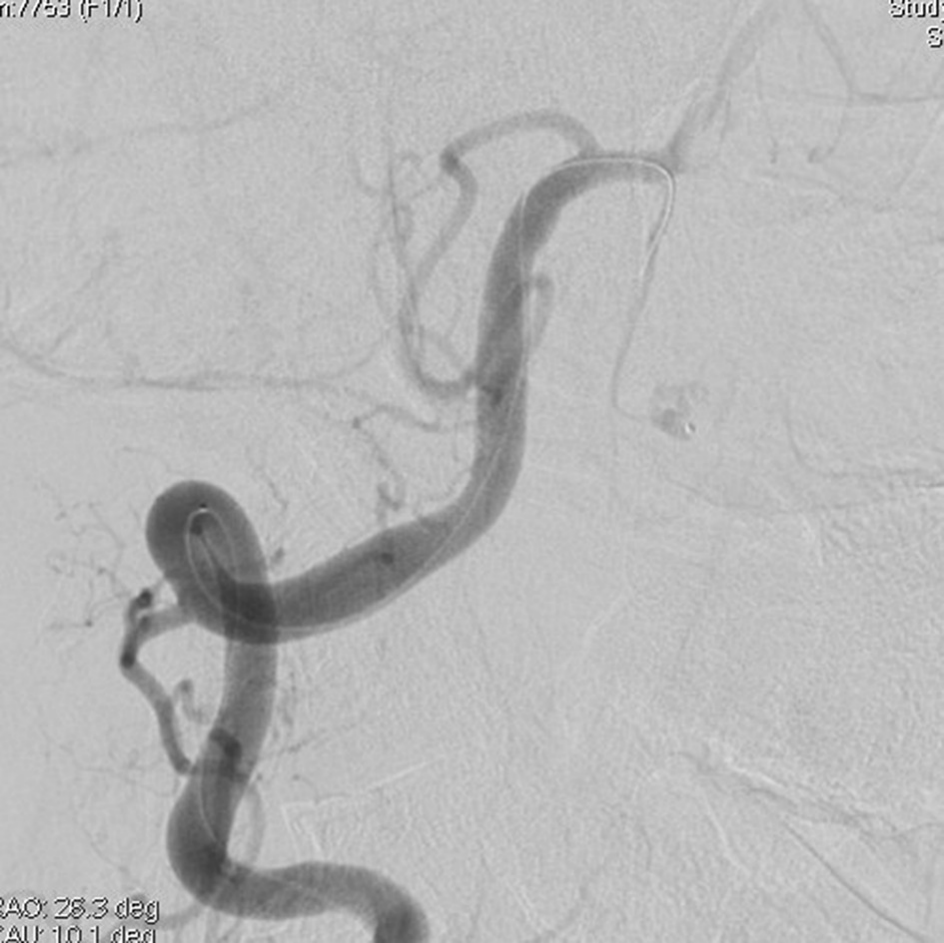
The Marathon® microcatheter (Covidien, Irvine, CA) is navigated to the aneurysm *via* the left anterior spinal artery.

**Figure 4.**
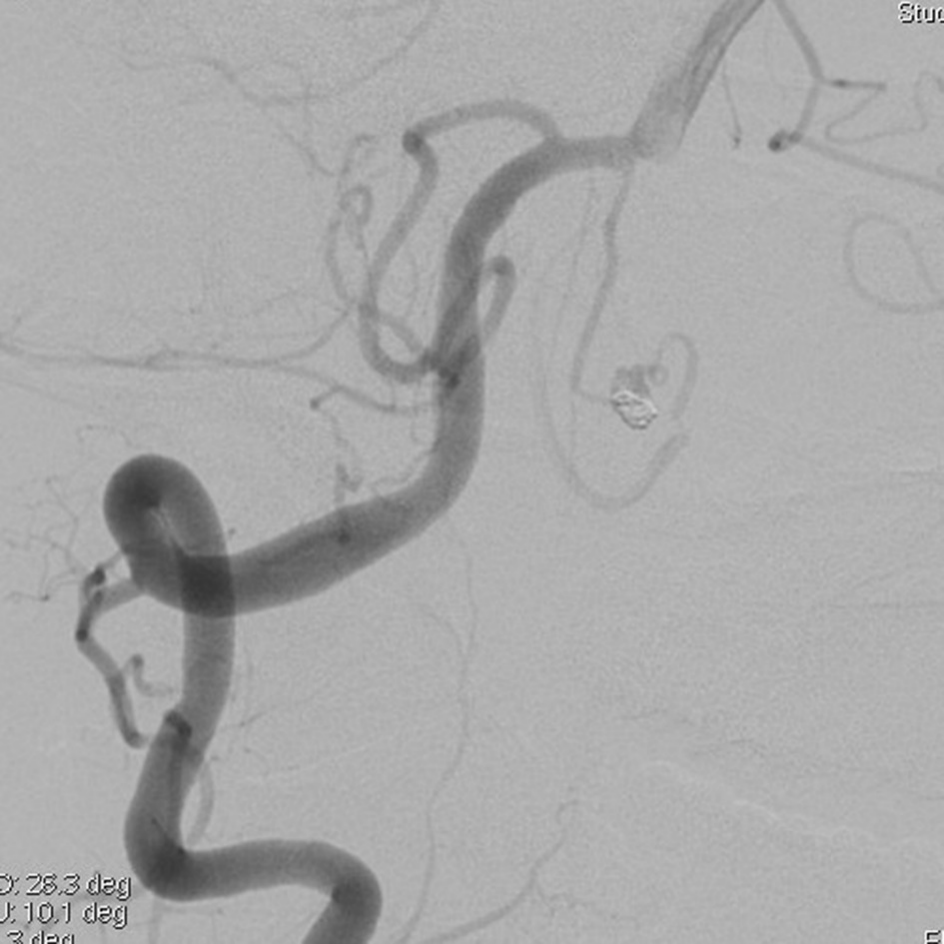
Selective angiography of the right vertebral artery after embolization. Complete elimination of the dome of the aneurysm is achieved. Blood flow in the vasa corona and the lateral spinal artery is preserved.

The patient’s aneurysm was embolized using detachable coils. Arterial access was obtained *via* a bilateral transfemoral approach. A 4-French diagnostic catheter (Radifocus; Terumo, Tokyo, Japan) was placed in the left vertebral artery *via* the left femoral sheath to visualize the aneurysm during the procedure. First, a Scepter XC balloon catheter™ (MicroVention Inc., Tustin, CA) was placed at the most distal segment of the right vertebral artery, just proximal to the vertebral union. Inflating the Scepter caused cessation of blood flow to the anterior spinal artery, but the left PICA was still well visualized by selective angiography through two small vessels from the left vertebral artery. Through this procedure, we confirmed collateral blood flow from the left vertebral artery to the left PICA. Following selective catheterization of the vasa corona *via* the left anterior spinal artery with a Marathon^®^ microcatheter (Covidien, Irvine, CA) and a Chikai 10^®^ (Asahi Intecc, Aichi, Japan) from the right vertebral artery (Figure 3), the aneurysm was occluded with four detachable microcoils (ED coil Extrasoft Type R; Kaneka Medix, Osaka, Japan). The final angiography (Figure 4) showed complete elimination of the dome of the aneurysm and preservation of blood flow from the vasa corona and the left PICA.

## Outcome, follow-up and discussion

The intra- and post-interventional courses were uneventful. The patient was discharged on day 7 after the intervention. Follow-up angiography 3 months after treatment showed disappearance of the aneurysm and preservation of the distal PICA. The patient’s diplopia, which was due to bilateral sixth cranial nerve palsy caused by the elevation of intracranial pressure after SAH, disappeared 6 months after treatment.

Isolated aneurysms involving spinal arteries are rare, and only one such case in a large series of more than 3000 spinal angiograms has been reported.^1^ Most of the aneurysms involving the spinal arteries have been associated with spinal cord arteriovenous malformations.^2^ Intracranial SAH due to the rupture of isolated spinal aneurysms is also a rare entity, and about 20 such cases have been reported until now.^3^ The aneurysms in these cases were located on the anterior spinal artery in seven, the radiculomedullary artery in nine and the posterior or lateral spinal artery in four cases, but no aneurysms were located on the vasa corona. Isolated aneurysms usually develop because of shear stress at the arterial junction and are less likely to develop in small vessels, such as the vasa corona. Therefore, most spinal artery aneurysms are formed by haemodynamic stress due to increased blood flow from the arteriovenous shunts.^2^

Here we have reported a case of an isolated aneurysm on the vasa corona along the medulla at the craniocervical junction. Haemodynamic stress following dysgenesis of the PICA induced the formation of an aneurysm. Such a case of an isolated aneurysm on the vasa corona has not been reported so far.

**Figure 5.**
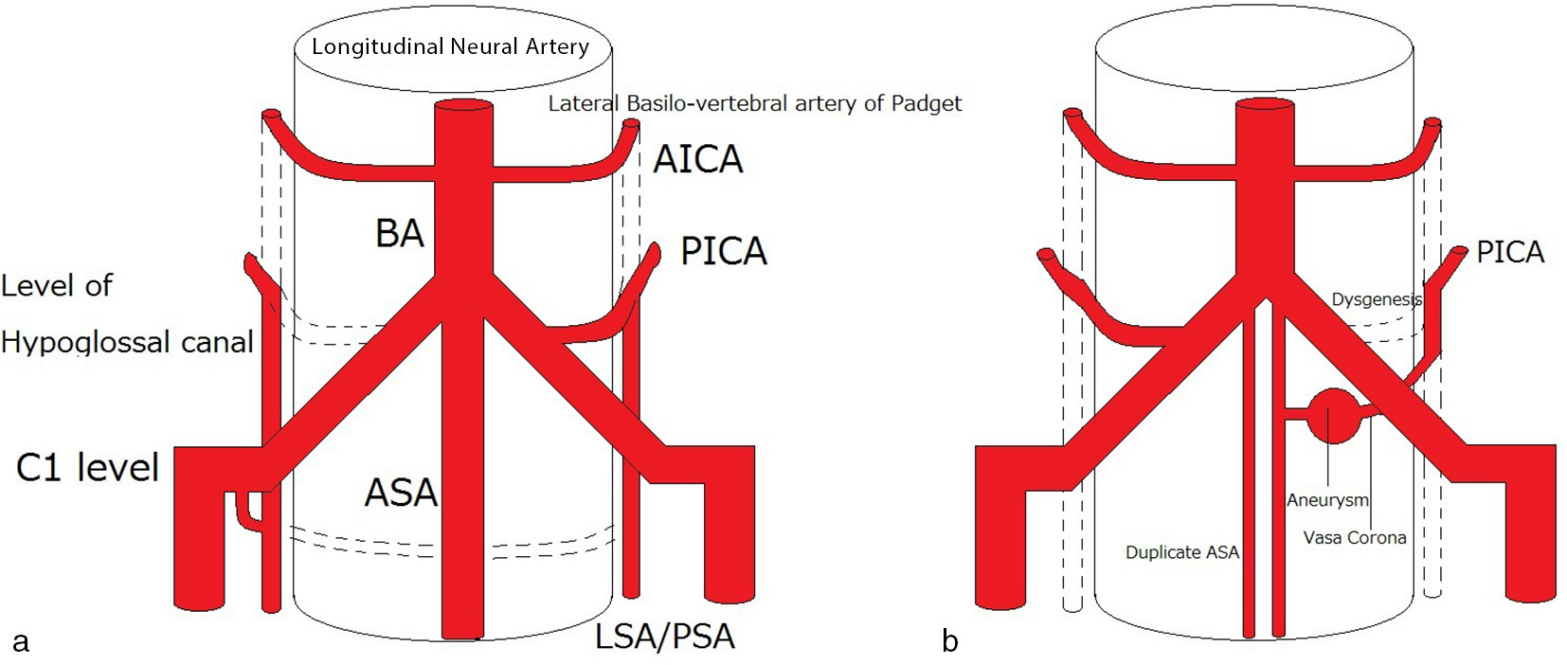
Schematic illustration of the arterial anatomy of the craniocervical junction. (a, normal case; b, the present case) In the present case, the aneurysm is located at the anastomosis (vasa corona) between the ASA and LSA. AICA, anterior inferior cerebellar artery; ASA, anterior spinal artery; BA, basilar artery; LSA, lateral spinal artery; PICA, posterior inferior cerebellar artery; PSA, posterior spinal artery.

In order to describe the pathogenesis of the aneurysm in the present case, a discussion about the embryology and anatomy of the arteries at the craniocervical junction is important. Embryologically, the proximal PICA derives from a hypertrophied radiculopial artery at the level of the hypoglossal canal.^4,5^ At an early stage, the PICA has numerous anastomoses between the nearby segmental and intersegmental arteries.^6,7^ Figure 5a illustrates these anastomoses at the craniocervical junction, among which one connection between the distal PICA and the vertebral artery become proximal PICA in the adult. This process explains the various anomalous origins and connections of the PICA noted in earlier reports.^7,8^

Lasjaunias et al^8^ showed that anatomic variations, such as the C1 and C2 origins of the PICA, duplication of the vertebral artery and the intradural course of the distal vertebral artery represented different sizes and connections between the lateral spinal artery, the PICA and the vertebral artery. Siclari et al^7^ also described anatomic variants of the distal vertebral artery and origin of the PICA. They classified the relationships between the PICA, posterior spinal artery (lateral spinal artery), vertebral artery and basilar artery into six groups.

PICA variations described by both Lasjaunias et al^8^ and Siclari et al^7^ did not include the PICA originating from the anterior spinal artery. PICA and the spinal arteries potentially have pial anastomoses, but the PICA originating from the anterior spinal artery and the vasa corona, instead of the vertebral artery, has not been reported and is a very infrequent route.

In the present case, the connection once formed between the left PICA and the left vertebral artery may have been lost in the later stages. In case of the agenesis of the connection between the PICA and the V4 segment of the vertebral artery, a firmer anastomosis would develop, such as the PICA arising from the vertebral artery at the C1 level. The loss of the proximal connection between the PICA and the vertebral artery in the present case resulted in the development of an aberrant route *via* the vasa corona (Figure 5b) and the tortuous small connection between the left PICA and the left vertebral artery.

The vasa corona is originally a small pial artery. Increased blood flow in the aberrant route might have caused haemodynamic stress on the arterial wall of the vasa corona, leading to formation of the aneurysm in the present case. A previous study had reported a case of an isolated lateral spinal artery aneurysm caused by haemodynamic stress owing to stenosis of the vertebral artery, the formation of which was similar to that of the present case.^9^

We treated the patient’s aneurysm with endovascular embolization as the aneurysm was ventral to the medulla at the craniocervical junction, and direct surgery on this aneurysm would have been difficult. Spinal aneurysms have been treated with direct surgical clipping in five, direct resection in five, endovascular therapy in four and observation in five cases.^3^ Direct surgery was more common in this entity because the spinal arteries, including the vasa corona, are small vessels and it is generally difficult to insert a microcatheter. In the present case, both the anterior spinal artery and the vasa corona were dilated owing to increased blood demand by the PICA, and the microcatheter successfully approached the aneurysm.

The most important aspect of treating the patient’s aneurysm was to preserve blood flow to the left PICA. We first temporarily inflated the balloon catheter immediately proximal to the vertebral union to stop blood flow from the right vertebral artery to the left PICA. Selective angiography from the left vertebral artery demonstrated firm enhancement of the left PICA through the small tortuous connection during cessation of blood flow from the right vertebral artery, indicating that blood flow of the left PICA would be preserved even if we occluded the parent artery.

Preservation of the vasa corona should have also been considered. In the present case, the aneurysm had ruptured and it was important to prevent re-rupture. Had we not embolized the aneurysm, it would have been trapped and the vasa corona could have been permanently occluded. In such a situation, the occlusion of the vasa corona could have caused infarction of the superficial surface of the medulla. In the present case, the vasa corona and PICA were preserved after selective embolization of the aneurysm in the vasa corona.

## Conclusions

Haemodynamic stress due to a vascular anomaly may cause the development of an isolated spinal artery aneurysm. We successfully treated a case of ruptured aneurysm with dysgenesis of the PICA by using endovascular techniques.

## Learning points

1. This is a rare clinical case and it is important to consider an aberrant vascular anomaly as the cause of this isolated spinal aneurysm.
2. Endovascular therapy can be used to treat an aneurysm in the vasa corona.

## Consent

Written informed consent was obtained from the patient for publication of this case report, including accompanying images.
